# Reactive Angioendotheliomatosis in Association with Ulcerative Colitis

**DOI:** 10.1155/2024/6505274

**Published:** 2024-05-29

**Authors:** R. Afiouni, G. Abadjian, F. Stephan

**Affiliations:** ^1^Dermatology Department, Hotel-Dieu de France University Hospital, Saint-Joseph University, Beirut, Lebanon; ^2^Pathology Department, Lebanese Geitaoui University Hospital, Beirut, Lebanon

## Abstract

Reactive angioendotheliomatosis (RAE) is a rare benign skin condition characterized histologically by the proliferation of dermal vessels and endothelial cells that occurs secondary to an underlying disease such as infections or lymphoproliferative disorders. To our knowledge, no previous cases of RAE associated with ulcerative colitis (UC) were reported in the literature. Therefore, we report the case of a 46-year-old man with a history of UC presenting with RAE confirmed on histopathology and immunostaining.

## 1. Introduction

Reactive angioendotheliomatosis (RAE) is a rare angioproliferative cutaneous condition, characterized histologically by the proliferation of dermal vessels and hyperplasia of endothelial cells within the lumina and around dermal vessels, with a benign evolution [1]. It can affect both men and women and occurs generally secondary to an underlying disease [2]. To our knowledge, no cases of RAE associated with ulcerative colitis (UC) or inflammatory bowel diseases were previously reported in the literature. Therefore, here, we report the first case of RAE occurring in a patient with a history of UC.

## 2. Case Presentation

**Figure 1:**
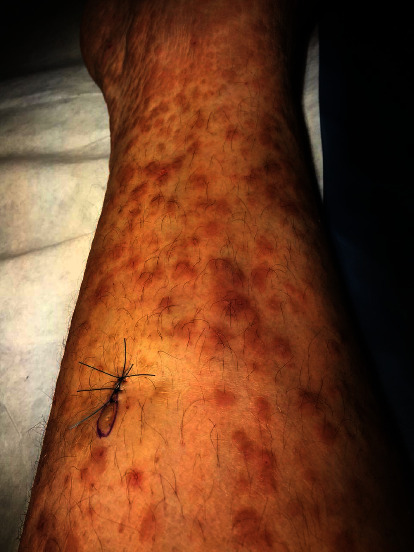
Clinical presentation, diffuse lichenification with well-demarcated violaceous and brown, indurated papules and plaques.

A 46-year-old man presented to our dermatology department for a few months history of diffuse pruritic skin lesions on his lower limbs. His past medical history consisted of a mild ulcerative colitis, well-controlled on mesalazine treatment, with a normal colonoscopy 6 months prior to his visit. He had no significant family history. Cutaneous examination revealed well-demarcated violaceous and brown, infiltrated, and indurated papules and plaques over both legs and thighs (Figure 1). No weight loss, fever, or gastrointestinal symptoms were reported. Differential diagnosis included leucocytoclastic vasculitis, Kaposi sarcoma, and dermatitis herpetiformis.

Blood tests revealed mild microcytic anemia (Hb 11 g/dL, MCV 73), leukocytosis (9.6 10^9^/L) with an elevated neutrophil count (8.31 10^9^/L), thrombocytosis (527 10^9^/L), slightly elevated erythrocyte sedimentation rate (ESR 54 mm/h) and C-reactive protein (CRP 9.7 mg/L), and a polyclonal hypergammaglobulinemia on serum protein electrophoresis. Further tests showed a negative antinuclear antibody (ANA): normal renal and liver enzymes tests except for a slightly elevated GGT, an elevated lactate dehydrogenase, and a normal TSH and negative viral serologies. A chest-abdomen-pelvis CT scan revealed a parietal thickening of the colon with a predominant right colon ectasia with multiple inflammatory mesenteric adenopathies consistent with colitis seen in inflammatory bowel diseases. Fecal calprotectin, a marker of disease activity, was elevated. All tests were in a favor of an active UC.

**Figure 2:**
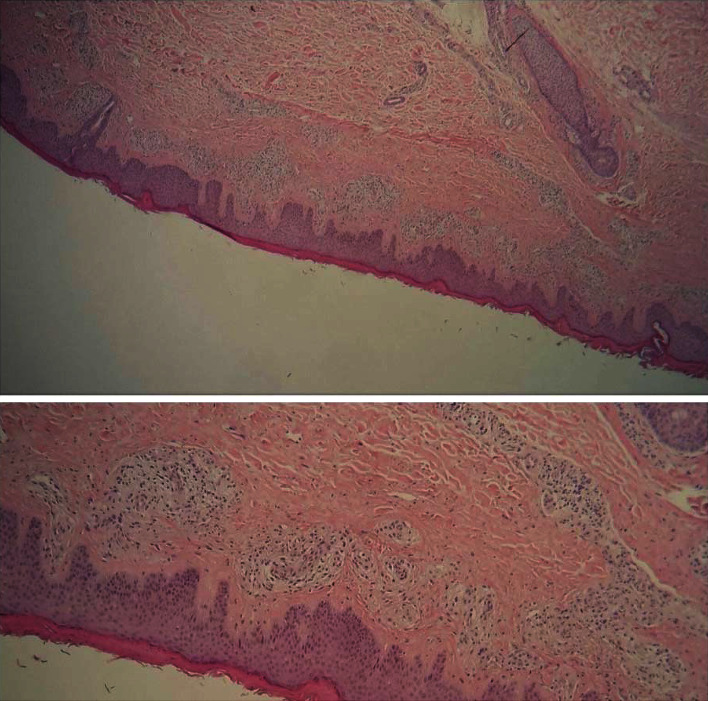
Proliferation of dermal capillaries with an intraluminal hyperplasia of endothelial cells and pericytes, with mononuclear infiltrate on HES.

**Figure 3:**
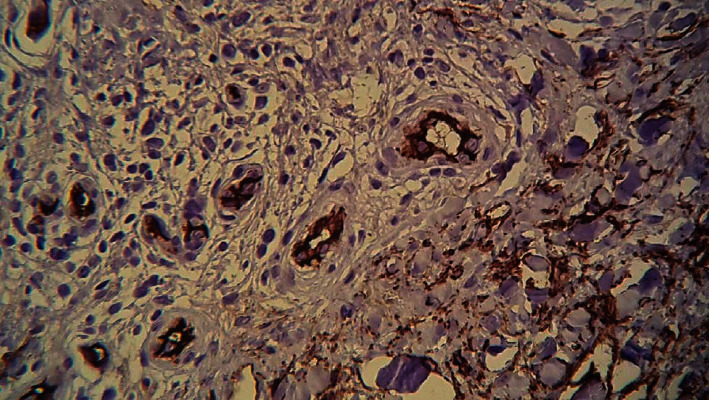
CD34 staining of proliferating endothelium.

A skin biopsy specimen was collected from a lesion on the right lower leg. Histopathologic examination showed a moderate acanthosis with compact hyperkeratosis, proliferation of dermal capillaries with an intraluminal hyperplasia of endothelial cells and a hyperplasia of pericytes, with mononuclear infiltrate and rare eosinophils (Figure 2). No intravascular thrombi and no cellular atypia were noted. The proliferating endothelium stained positive for CD34 and CD31 (Figure 3). D2-40 and HHV-8 were negative. These histologic findings were compatible with a reactive angioendotheliomatosis.

The patient received treatment for his colitis including systemic steroids with a close follow-up. Skin emollients were also given with a subsequent improvement then remission of his lesions.

## 3. Discussion

Reactive angioendotheliomatosis (RAE) is a rare benign vascular and cutaneous condition occurring secondary to an underlying disease but with an uncertain pathogenesis [2]. It is considered that vascular injury or occlusion from the deposition of various agents results in local hypoxia, then in the release of angiogenic factors which induce vascular proliferation [3].

Clinically, patients can present with a wide range of features, including asymptomatic, pruritic, or painful, erythematous to violaceous macules, plaques, or papules, that can become ulcerated or necrotic. Lesions are primarily located on the limbs, but the face and trunk can also be affected [4]. Differential diagnosis may include mycosis fungoides, sarcoidosis, Kaposi's sarcoma, acroangiodermatitis, systemic lupus erythematosus, leucocytoclastic vasculitis, and dermatitis herpetiformis [5].

RAE occurs secondary to an underlying disease including systemic infections such as endocarditis and hepatitis, autoimmune diseases such as rheumatoid arthritis, sarcoidosis and antiphospholipid syndrome, arteriovenous shunt, end-stage renal disease, hematologic, and solid malignancies such as breast cancer, peripheral vascular diseases, and hypercoagulable conditions [1, 2, 6–9]. Chen et al. described a case of RAE associated with POEMS syndrome [10]. A case of RAE was also reported secondary to propylthiouracil treatment for hyperthyroidism. The authors raised the question of the possible role of ANCA auto-antibodies in the pathogenesis of RAE in this case [11]. Hence, laboratory studies may reveal the majority of cases: anemia, leukocytosis, low platelet count, elevated LDH levels, and elevated erythrocyte sedimentation rate [2]. RAE with an underlying ulcerative colitis, as seen in our case, has not been described in the literature, which could be added to the list of systemic diseases in RAE. The inflammation associated with UC and subsequent vascular injury may have played a role in the occurrence of RAE. ANCA auto-antibodies, which are commonly described in UC, could also participate in the pathogenesis in this case [11]. In addition, in our patient, RAE lesions were the only clinical sign which revealed an underlying active UC that was later confirmed on laboratory tests.

The diagnosis of RAE is confirmed on skin biopsy which reveals characteristic features consisting of proliferation of dermal vessels and hyperplasia of endothelial cells within the lumina and around the vessels [1]. On immunohistochemistry, the intravascular proliferating cells are positive for CD31 and CD34, as detected in our patient [12].

RAE is a benign cutaneous reactive angiomatosis that must be distinguished from the malignant intravascular large B cell lymphoma as they both can have a similar clinical presentation. Absence of cellular atypia and a lack of T- or B-cell on immunostaining are in a favor of RAE in this case [5].

In addition, leucocytoclastic vasculitis (LCV), an immune-complex mediated disease, should be kept in mind as a differential diagnosis because early treatment is necessary. LCV may rarely occur in the setting of UC, before, during, or after the diagnosis [13]. It presents clinically with palpable purpura, erythematous plaques or macules, or bullae, typically on the lower extremities but can also occur on the upper extremities [13]. Histopathologically, it is characterized with an inflammation of postcapillary venules and a neutrophilic infiltration [14].

Finally, RAE is self-limiting condition. Therefore, no specific treatment is used. It is usually managed by treating the underlying systemic disorder but recurrences remain possible [15, 16]. Systemic steroids have been used [16]. In our patient, systemic steroids have been given to treat his active UC with a subsequent remission of RAE lesions.

Ulceration secondary to RAE is usually painful and requires faster therapy. Pulsed dye laser (PDL) is an option to treat those patients with a significant and rapid response [8]. Bridgewater et al. suggested that PDL downregulates vascular growth factors by a systemic anti-inflammatory effect in addition to targeting a direct necrosis of vessels [8]. Treatment with topical timolol can be an option via its vasoconstriction effect in addition to the inhibition of vascular endothelial growth factors. In fact, Bhatia et al. described a complete resolution of lesions of post-traumatic RAE after three time daily application of timolol maleate 0.5% ophthalmic solution for 6 weeks [17, 18].

In conclusion, although RAE is a benign condition with a polymorphous presentation, it should be kept in mind as it could reveal an underlying systemic condition or also a disease activity such as with UC which we describe its association with RAE for the first time in the literature. Hence, it is important to perform the appropriate laboratory and imaging investigation when RAE diagnosis is made.
